# Supplementary material for: A Flexible Supercapacitor Based on Niobium Carbide MXene and Sodium Anthraquinone-2-Sulfonate Composite Electrode
Source: Micromachines (Basel). 2023 Jul 28;14(8):1515. doi: 10.3390/mi14081515 (PMC10456233; doi:10.3390/mi14081515)
Supplement: Supplementary file 1 [file micromachines-14-01515-s001.zip › micromachines-2493420-supplementary.pdf]

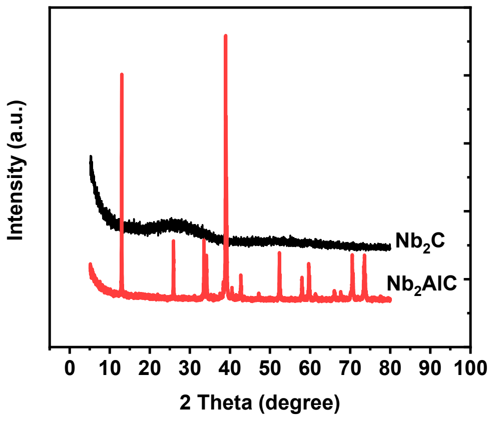

Figure S1. XRD patterns of Nb_2C nanosheets and Nb_2AlC powder.

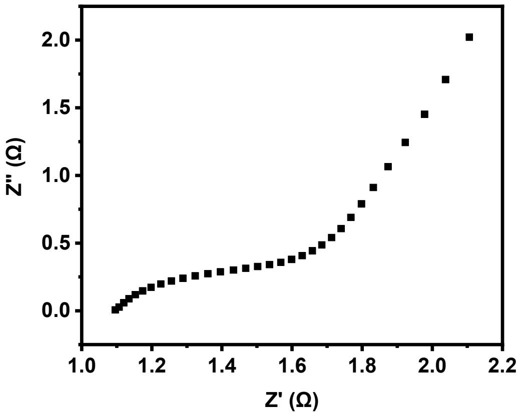

Figure S2. Nyquist plots of the Nb_2C–AQS nanocomposite-modified electrode in 10 mmol·L^{-1} $K_4Fe(CN)_6$ and $K_4Fe(CN)_6$ (molar ratio 1:1) from 1–10 mHz at 10 mV sinusoidal signal.

The areal specific capacitance (C_s) of the electrodes were obtained from the CV curves [1]:

$$C_A = \left(\frac{1}{2} \int idV \right) / \left(A \times \Delta V \times v \right),$$

where C_A represents the specific capacitance (F·cm^{-2}), $\int idV$ is the integrated area of the CV curve, ΔV is the scanning potential window (V), and v is the scan rate (V·s^{-1}). The CV curves consist of oxidation and reduction curves corresponding to charge/discharge processes. Thus, the integrated area of the nearly symmetrical CV curves (multiplied by ½) was used to calculate the specific capacitance.

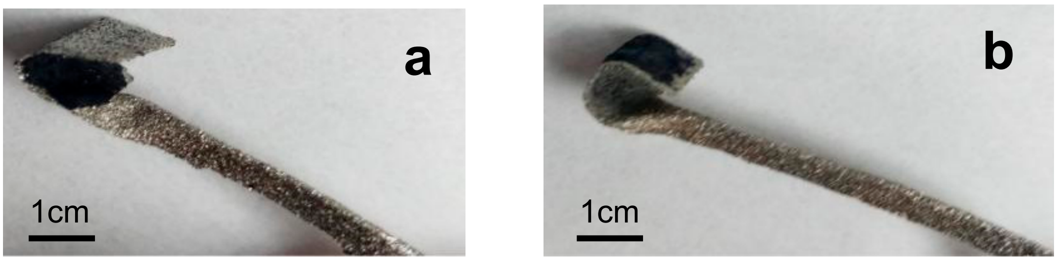

Figure S3. Two bending modes of the Nb_2C–AQS based SC: (a) concavely bent, and (b) convexly bent.

The specific capacitance of the resulting SCs was also derived from the GCD curves of the Nb_2C–AQS composite:

$$C_A = T / (\Delta V \times i \times 2),$$

where C_A represents the specific capacitance (F·cm^{-2}), T represents the discharge time of a cycle of constant current charge and discharge, ΔV is the scanning potential window (V), and i is the current density (Å·cm^{-2}).

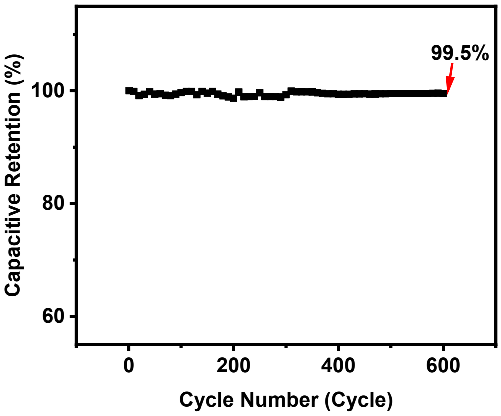

Figure S4. Capacitance retention at a current density of 15 mA cm^{-2} for the Nb_2C–AQS based micro-SC in 0.1 mol L^{-1} Na_2SO_4.

Reference:

1 Wang, G.X.; Babaahmadi, V.; He, N.; Liu, Y.; Pan, Q.; Montazer, M.; Gao, W. Wearable supercapacitors on polyethylene terephthalate fabrics with good wash fastness and high flexibility. Journal of Power Sources, 2017; 367(nov.1):34-41.
